# Stunting Among Low-Income Families in Indonesia: Is Mother’s Employment a Risk Factor?

**DOI:** 10.34172/jrhs.7450

**Published:** 2025-06-10

**Authors:** Ratna Dwi Wulandari, Agung Dwi Laksono, Yuly Astuti, Ratu Matahari, Nikmatur Rohmah, Rohani Budi Prihatin, Frima Elda

**Affiliations:** ^1^Department of Health Policy and Administration, Faculty of Public Health Science, Universitas Airlangga, Surabaya, Indonesia; ^2^National Research and Innovation Agency Republic of Indonesia, Jakarta, Indonesia; ^3^Department of Reproductive Health, Faculty of Public Health Science, Universitas Ahmad Dahlan, Yogyakarta, Indonesia; ^4^Department of Pediatric Nursing, Faculty of Health Sciences, Muhammadiyah University of Jember, Jember, Indonesia; ^5^Center for Legislative Analysis of Indonesian Parliament, Jakarta, Indonesia; ^6^Department of Nutrition, Faculty of Public Health, Universitas Andalas, Padang, Indonesia

**Keywords:** Poverty, Maternal employment, Stunting, Employed mother, Public health nutrition

## Abstract

**Background:** It is widely believed that poverty is a significant factor in causing stunting, and parental habits can also play a role. In this context, households with employed mothers are often suspected of increasing the risk of stunting in children. This study aimed to examine the role of maternal employment status in stunted children among low-income families in Indonesia.

**Study Design:** A cross-sectional analysis.

**Methods:** In general, 47021 children were investigated in this study. The children’s nutritional health and maternal work status were used as the outcome and variables, respectively. Seven control variables were evaluated, including residence type, marital status, mother’s age, education level, child age, antenatal care (ANC), and gender. Finally, a binary logistic regression analysis was employed, ensuring the validity and reliability of the results.

**Results:** Overall, 19.0% of Indonesian children under two from low-income households experienced stunted growth. Meanwhile, 23.5% of mothers were employed in low-income homes. Regarding nutritional status, the findings revealed that employed mothers had stunted kids, which is slightly higher than that in unemployed mothers. More importantly, it was found that unemployed mothers were 1.022 times more likely to have stunted kids than employed mothers (95% confidence interval: 1.015–1.030), underscoring the crucial role of maternal employment in child nutrition.

**Conclusion:** Maternal employment status played a role in having stunted children among low-income families, and unemployed mothers were a risk factor for having this type of child. Empowering unemployed mothers through job opportunities, financial support, and access to childcare and nutrition programs can help reduce child stunting in low-income families.

## Background

 The World Health Organization (WHO) defines stunting as chronic malnutrition in which a child’s height-for-age Z-score is less than minus two standard deviations.^1^ Early stunting causes physical and clinical problems for the child.^2^ It increases the risk of infectious illness and morbidity in the first five years of life. Stunted children may gain weight quickly after two, leading to obesity or overweight later in life.^2^ According to research, stunting harms cognitive development, scholastic performance, and adult economic productivity.^3,4^ It can also cause degenerative diseases such as heart disease, stroke, and diabetes.^5^

 In 2020, the United Nations Children’s Fund (UNICEF), WHO, and the World Bank found that 24.1% of Southeast Asian children under five were stunted.^6^ Indonesia has one of the highest stunting rates in this region, second only to 33.1% in East Timor.^7^ Although Indonesia has prioritized stunting reduction since 2017, progress has been gradual. Between 2012 and 2020, Indonesia’s stunting rate dropped by 2.7%, from 34.5% to 31.8%.^7^ This decline is less than the regional average of 3.6% and much less than the 5.8% decrease in five target nations (Lao PDR, Cambodia, the Philippines, Myanmar, and Vietnam).^7^

 Several factors cause childhood stunting, according to the WHO framework. Suboptimal feeding by mothers can increase stunting risk.^8,9^ Infection, mainly recurring infection and vaccination status, as well as societal variables, such as rural living, poor drinking water, and inadequate sanitation, all contribute to stunting.^10,11^ Numerous studies have linked stunting to poor socioeconomic situations, such as reduced household income, maternal work status, maternal health, and child features, including age and gender.^12-14^

 The link between stunting and socioeconomic factors is growing.^15,16^ Various studies have shown that disadvantaged children are more likely to be stunted.^12-14^ Population-based surveys in African countries regularly reveal that children from low-income families are more likely to be stunted due to the limited availability of sufficient nutrition and optimal feeding habits.^17^ Poor housing increases the risk of infection, inadequate child care, and limited access to needed healthcare.^10^ Poor mothers also have lower education levels, which might lead to baby-feeding ignorance.^18^

 Employed mothers are more likely to have stunted children than unemployed mothers, according to previous studies.^9,19^ Due to work responsibilities, employed mothers have less time for good childcare, which impacts exclusive breastfeeding, early weaning, and incorrect complementary feeding.^9^ For instance, a study in Ghana reported that work pressure and inadequate workplace facilities hinder optimal breastfeeding, leading to stunting.^20^ The Indonesian Health Law, Number 36 of 2009, requires the government and the public to provide enabling facilities for exclusive breastfeeding at work, but employed mothers often lack access to such facilities.

 This study is noteworthy because socioeconomic determinants have been linked to stunting, although maternal employment status in low-income families is poorly understood.^14,19,21^ Employed mothers may have more financial means yet struggle to care for their children; thus, this gap must be closed. This study may suggest supporting employed women with maternity leave, quality daycare, and community-based nutrition and health programs. These findings may help design more effective stunting therapies for low-income Indonesian households. Considering the background, the study seeks to examine the role of employment of mothers with stunted kids among low-income families in Indonesia.

## Methods

###  Study design and data source

 Additional data from the 2022 Indonesian National Nutrition Status Survey was used for this study. Indonesia’s Ministry of Health conducted a cross-sectional study. All Indonesian-born families with low income and children under two were also surveyed. The mother was the respondent in this child-centered study. The survey collected a weighted sample of 47 021 people under two conditions using multistage cluster random sampling. The poll had a 91.4% response.^22^

###  Setting

 This study involved nationwide low-income families. Property wealth quintiles were considered to determine a household’s socioeconomic position using principal component analysis. The survey assessed the quantity and type of home goods. In addition, the study examined televisions, bicycles, autos, and demographics to predict wealth. Moreover, it evaluated the significant buildings, lavatories, and potable water sources. The scores were calculated using principal component analysis. Overall, 20% of the population was surveyed, and their household scores were summed up to establish wealth quintiles. Five groups were formed after subdividing the quintiles. The social class was divided into five quintiles, representing the poorest (quintile 1), the poorer (quintile 2), the middle (quintile 3), the wealthier (quintile 4), and the richest (quintile 5). Low-income families refer to households that fall within quintiles 1 and 2.^23^

###  Outcome variable

 The nutritional status (stunting) was the dependent variable in the study. Stunting was categorized into either typical ( ≥ -2.0 standard deviation) or severe ( < -2.0 standard deviation) types. The WHO growth standards provide the height indicator, known as the z-score or height departure from the average size, utilized to assess a child’s nutritional status, depending on their age or height at a certain point in time.^18^

###  Exposure variables

 The study used maternal employment status as an exposure variable. Maternal employment is the mother’s acknowledgment of whether she works or not. The study split maternal employment into unemployed and employed statuses.

###  Control variables

 The kind of habitation, mother’s age, education level, marital status, antenatal care (ANC) during pregnancy, age of children, and gender were the control variables examined in this study. Both urban and rural types of dwelling were included, and seven age groups of mothers were identified based on the data ( < 19, 20–24, 25–29, 30–34, 35–39, 40–44, and > 45). Maternal marriage was classified into married, divorced, or widowed groups. Moreover, four levels of maternal education were recognized, including elementary, middle, high school, and high school levels.

 ANC services, both performed and unperformed throughout pregnancy, were also included in the investigation. The children were split into 0–11 and 12–23 age groups (in months). However, no distinction between men and women was made in this study.

###  Data analysis

 First, the chi-square test was applied, and then, a collinearity test ascertained the presence of a statistically significant correlation between independent variables. Next, a binary logistic regression analysis was conducted, and finally, statistical computations were performed using the IBM SPSS Statistical software, version 26.

 In addition, a distribution map of employed mothers and stunted children was created by province in Indonesia using ArcGIS 10.3 (ESRI Inc., Redlands, CA, USA). Statistics Indonesia supplied a shapefile with administrative border polygons for the study.

###  Ethical approval and consent to participate

 Secondary data for the study were obtained from the Indonesian National Nutritional Status Survey in 2022. The National Ethics Commission has declared the study “exempt” (the notification letter is attached). After obtaining written informed consent, the Indonesian Ministry of Health gathered information for the 2022 Indonesian National Nutritional Status Survey. Participants signed informed permission forms to stress the voluntary and private character of the data-collecting procedure. These Internet resources were provided to researchers by the Indonesian Ministry of Health (https://layanandata.kemkes.go.id/).

## Results

**Figure 1:**
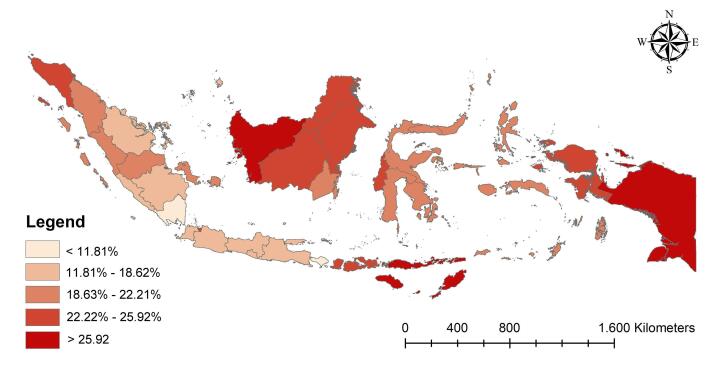
The prevalence of stunted growth among children under two in low-income households in the provinces of Indonesia. *Source*. Visualization by the authors

 The results demonstrated that 19.0% of Indonesian children under two from low-income households experienced stunted growth. Additionally, at the national level, 23.5% of mothers were employed in low-income households in Indonesia. Figure 1 shows a map of stunted growth in low-income Indonesian children under two, displaying that Papua and Kalimantan had the most stunted children.

**Figure 2:**
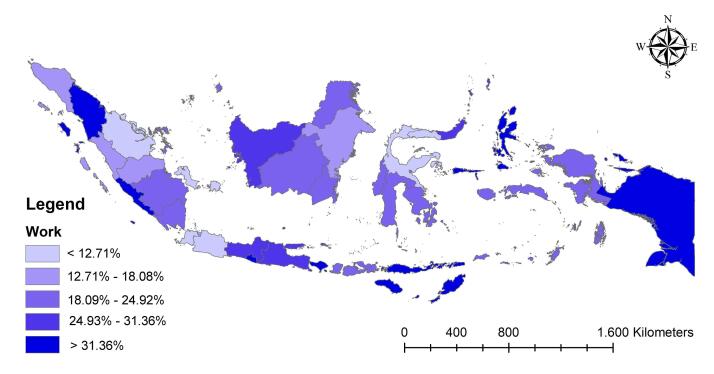
The distribution map of employed mothers among low-income families in the provinces of Indonesia. *Source*. Visualization by the authors

Figure 2 depicts a map of employed mothers in low-income Indonesian households. According to the data, Papua, Nusa Tenggara, and Maluku had more employed mothers. Overall, eastern Indonesia was more vulnerable than other regions.

Table 1 presents descriptive data regarding the job status of mothers and the nutritional condition of Indonesian children under the age of two in low-income households. Regarding nutritional status, the results revealed that employed mothers had stunted kids, which is slightly higher than that in unemployed mothers. According to residence type, the number of employed mothers in rural areas was almost twice that of those in urban areas. Based on maternal age, the 30–34 age group had the highest proportion in the employed mothers’ group. Moreover, mothers with senior high school education had the highest ratio of employed mothers’ kind.

**Table 1:** The correlation between the job status of mothers and the nutritional well-being of children under two in low-income households in Indonesia (*N* = 47 021)

**Variables**	**Unemployed ** **(n=32407)**		**Employed ** **(n=14614)**		***P*** ** value**
	**Number**	**%**	**Number**	**%**	
Nutritional status					0.048
Normal	25 945	81.0	37 586	80.9	
Stunted	6462	19.0	9435	19.1	
Residence type					0.001
Urban	13 730	45.8	4578	37.8	
Rural	18 677	54.2	10 036	62.2	
Maternal age (year)					0.001
≤ 19	1209	3.2	267	1.6	
20-24	6336	19.4	1852	13.1	
25-29	9050	28.8	3826	26.0	
30-34	7772	24.5	4275	28.8	
35-39	5273	15.9	2901	20.2	
40-44	2300	6.9	1227	8.3	
≥ 45	467	1.3	266	2.1	
Maternal education level					0.001
Primary school	10 862	35.2	4200	31.0	
Junior high school	9221	30.5	3317	25.2	
Senior high school	11 421	32.4	5489	34.6	
College	903	2.0	160	9.1	
Maternal marital status					0.001
Married	31 962	98.8	14 210	97.0	
Divorced/Widowed	445	1.2	404	3.0	
ANC during pregnancy					0.001
No	2993	6.1	2116	10.4	
Yes	29 414	93.9	12 498	89.6	
Kid’s age (months)					0.001
0-11	15 844	48.3	6718	45.0	
12-23	16 563	51.7	7896	55.0	
Kid’s gender					0.001
Boy	16 588	51.1	7591	50.7	
Girl	15 819	48.9	7023	49.3	

*Note*. ANC: Antenatal care.

 Based on the results (Table 1), married mothers dominated both employment status groups regarding maternal marital status. According to ANC, unemployed mothers had a higher proportion than employed mothers performing ANC. Based on the kids’ age, those in the age group of 12–23 years had a higher proportion than those in the age group of 0–11 in both kinds of maternal employment. Furthermore, boys had a higher ratio in all maternal employment statuses.

 The research was conducted following collinearity studies. The trial findings showed that the independent variables did not display collinearity. The data indicated that the variance inflation factor values of all variables were below 10.00. Additionally, the mean tolerance values for all variables were statistically significant, surpassing 0.10. The multicollinearity test was used to determine the presence of a significant correlation between two or more independent variables in the regression model.

 As previously mentioned, this study examined the nutritional status of Indonesian children under two years old from low-income families using binary logistic regression analysis. The results (Table 2) revealed that unemployed mothers were 1.022 times more likely to have stunted kids than employed mothers (95% confidence interval [CI]: 1.015–1.030). As regards the residence type, mothers in urban areas were 1.039 times more likely to have stunted children than those in rural areas. Further, all maternal ages were more likely than ≥ 45 to have stunted kids (1.032–1.046).

**Table 2:** The nutritional status (stunting) of Indonesian children under two years old from low-income families in Indonesia using binary logistic regression analysis (*N* = 47,021)

**Variables**	**Adjusted OR (95% CI)**	***P *** **value**
Maternal employment		
Employed	Ref.	
Unemployed	1.022 (1.015–1.030)	0.001
Residential area		
Urban	Ref.	
Rural	1.039 (1.032–1.046)	0.001
Maternal age		
≤ 19	1.723 (1.669–1.779)	0.001
20-24	1.375 (1.338–1.412)	0.001
25-29	1.398 (1.361–1.436)	0.001
30-34	1.408 (1.370–1.446)	0.001
35-39	1.333 (1.297–1.369)	0.001
40-44	1.339 (1.302–1.378)	0.001
≥ 45	Ref.	
Maternal education		
Primary school	1.436 (1.410–1.463)	0.001
Junior high school	1.254 (1.231–1.277)	0.001
Senior high school	1.145 (1.125–1.167)	0.001
College	Ref.	
Marital status		
Divorced/widowed	Ref.	
Married	1.016 (0.992–1.041)	0.182
Antenatal care		
Yes	Ref.	
No	1.492 (1.475–1.509)	0.001
Kids’ age (months)		
0-11	Ref.	
12-23	3.317 (3.294–3.339)	0.001
Kids’ gender		
Girl	Ref.	
Boy	1.385 (1.377–1.394)	0.001

*Note*. OR: Odds ratio; CI: Confidence interval.

 Based on maternal education, the obtained data (Table 2) indicated that the possibility of having stunted children was higher when the maternal education was lower. Concerning ANC during pregnancy, mothers who did not perform ANC were 1.492 times more likely to have stunted kids than those who performed ANC during pregnancy (95% CI: 1.475–1.509). Kids aged 12–23 were 3.317 times more likely to be stunted than those aged 0–11 (95% CI: 3.294–3.339). Furthermore, based on kids’ gender, boys were 1.385 times more likely to be stunted than girls (95% CI: 1.377–1.394).

## Discussion

 Our findings confirmed that unemployed women had more stunted children. Even though it is small, the employment of mothers prevents stunting. Nonetheless, the study results are intriguing and thus call for further investigation. Maternal employment may protect against stunting through numerous mechanisms, including higher household income, maternal education, and healthcare and nutrition access. First, employed mothers contribute monetarily to their households, improving food security, diet diversity, and living conditions for child growth and development. Higher income can help families get regular checkups, vaccines, and early child malnutrition treatment. Employed mothers may also have more schooling or job exposure to health and nutrition information, which can improve their knowledge of child feeding, hygiene, and disease prevention. Employment may also boost a mother’s household decision-making power, prioritizing child health spending and improving care. Finally, maternal employment can provide paid maternity leave and breastfeeding assistance programs to enable sustained maternal care during a child’s essential early years. These indicators show how maternal employment might help low-income children avoid stunting. Based on the data obtained from Gondar city, Bangladesh, and Bale Robe city, Ethiopia, children of employed mothers were more likely to be stunted.^20,24,25^ The study results are in line with those of studies on urban Indonesian children whose mothers worked, who were more likely to be stunted than rural children.^14^ Conversely, mothers employed in rural regions exhibited a greater likelihood of having toddlers who were stunted or severely stunted than those who were unemployed.^19^ The data suggest that employed women represent a demographic susceptible to child stunting.

 On the other hand, the findings revealed that kids’ age plays the most significant role in their nutritional status. Kids aged 12–23 were 3.317 times more likely to be stunted than those aged 0–11, indicating that younger children are more nutritionally fulfilled than older children. The mothers’ caring role for younger children is also more prominent than their role for older children. The results of this study conform to those of studies performed in 94 low- and middle-income countries. The prevalence of stunting is higher in older children up to around 28 months of age. This may be due to longer exposure times and accumulated adverse exposure to nutritional deficiencies and infections.^26^ Similarly, a study in Angola found evidence that the risk of stunting increased sharply in the second, third, and fourth years of life.^27^ These results are also consistent with previous findings, highlighting that stunting occurs more frequently in children aged 12–23 months compared to children < 12 months.^16^ These findings indicate that children over 12 months require more serious attention in meeting growth and development needs, and this age group is more susceptible to stunting.

 Regarding residence type, mothers in urban areas were more likely to have stunted children than those living in rural areas. Studies showed that the disparity between urban and rural areas in the prevalence of stunting among children can vary, reflecting inequalities in access to nutrition, healthcare, and other socioeconomic factors.^14^ Despite better access to healthcare and education in urban areas, economic disparities, overcrowding, poor sanitation, and limited access to nutritious food might contribute to higher stunting rates.^16,28,29^ Mothers who work in urban areas have a higher proportion of stunted children than those in rural areas. Increasing income from employed mothers is implicated in the higher consumption of processed and high-fat foods, contributing to malnutrition.^14^ Urban poverty can lead to malnutrition, as families may struggle with the high cost of living, leading to insufficient dietary intake and poor health outcomes for children.^30^

 Meanwhile, all maternal ages were more likely to have stunted kids than ≥ 45. This could be due to various factors, including biological, socioeconomic, and environmental influences. Teenage mothers or mothers in their twenties and thirties might lack experience, education, or resources to provide optimal nutrition and healthcare for their children, increasing the risk of stunting. Younger mothers might be less economically stable, leading to inadequate access to nutritious food and healthcare. They might have lower levels of education, affecting their knowledge about proper nutrition and child-rearing practices.^31^ Mothers, as caregivers, make all the decisions about healthy feeding practices, including breastfeeding.^19^ Previous research reported a contradictory finding, demonstrating that older maternal age was associated with a higher risk of stunting, while the hypothesis was that younger maternal age could increase the risk.^12^

 Based on maternal education, the possibility of having stunted children is higher when the maternal education is lower. Mothers with higher education levels are more likely to be informed about proper nutrition, childcare practices, and the importance of a balanced diet, leading to better health outcomes for their children.^13^ In addition, more educated mothers are generally better informed about proper nutrition and healthcare practices for their children. Further, educated mothers are more likely to utilize healthcare services, which can prevent and treat conditions that lead to stunting.^16^ Education often correlates with higher socioeconomic status, which can provide better access to nutritious food and healthcare resources. Education empowers women to make informed decisions about their health and their children’s health.^14^ Likewise, higher education often correlates with better job opportunities and higher income, enabling mothers to afford nutritious food and quality healthcare for their children. The previous research revealed that a better education level was a protective factor against employed mothers having stunted toddlers.^32^

 Regarding ANC during pregnancy, mothers who did not perform ANC were more likely to have stunted kids than those who did. ANC during pregnancy is crucial for the health and development of children. The WHO’s guidelines on ANC emphasize the importance of routine care for pregnant women to ensure positive perinatal and maternal outcomes.^33^ These guidelines recommend a comprehensive package of services that includes nutritional interventions, disease prevention, and early detection of pregnancy-related conditions. Studies have indicated that mothers who do not receive ANC are at a higher risk of having children who are stunted.^33^ ANC visits allow healthcare providers to offer critical interventions that promote healthy growth and prevent stunting. This includes nutritional advice, supplements, and the child’s growth monitoring.^34^ By ensuring regular ANC visits, mothers can receive the support and care needed to reduce the likelihood of stunting and other developmental issues in their children.

 Furthermore, based on kids’ gender, boys were more likely to be stunted than girls, which is consistent with the results of research conducted in Indonesia, indicating that the risk of stunting is higher in sons than daughters.^35^ Additionally, a meta-analysis study regarding the gender of infants with malnutrition found that there was a greater propensity for sons than for daughters.^36^ Likely, a son’s increased susceptibility to an infection that results in severe nutritional loss and weight loss is correlated with a higher tendency to stunt males than daughters.^37^

## Strengths and Limitations

 The report draws national findings from careful data analysis. This study only used survey components as extra data. Previous research examined significant variables that this study ignored. Height, weight, anemia, diarrhea, and pregnant agricultural production can affect stunting in children.^38,39^ This study had a cross-sectional design, implying that the study results are not generalizable to all cases; we can only conclude that there is a relationship between stunting and the independent variable. Additionally, the study’s quantitative approach ignores Indonesia’s cultural traditions. Other studies on offspring, restricted food intake, child-rearing, and diet have influenced the interrelated discoveries.^40^

**Highlights**

- Almost 19.0% of Indonesian children under two from low-income households experienced stunted growth.
- Maternal employment status had a role in stunted children among low-income families.
- Mothers’ unemployment was a risk factor for having stunted children.
- Seven control variables, including residence type, mother’s age, marital status, education level, antenatal care (ANC), child age, and gender, were associated with stunting.

## Conclusion

 Based on the results, maternal employment status had a role in stunted children. The results further revealed that unemployed mothers are a risk factor for having stunted children. Moreover, it was found that kids’ age plays the most significant role in their nutritional status. Kids aged 12–23 were 3.317 times more likely to be stunted than kids aged 0–11.

 Based on the findings, policies should focus on empowering unemployed mothers through job opportunities, financial assistance, and access to affordable childcare to reduce the risk of stunting. Additionally, targeted interventions for children aged 12–23 months, such as improved nutrition programs, growth monitoring, and parental education on complementary feeding, are crucial. Strengthening these support systems will help improve children’s nutritional status and overall health outcomes.

## Acknowledgments

 The author would like to thank the Ministry of Health of the Republic of Indonesia for providing data from the 2022 Indonesian National Nutrition Status Survey.

## Competing Interests

 The authors declare that they have no conflict of interests.

## Ethical Approval

 Secondary data for the study were collected from the 2022 Indonesian National Nutritional Status Survey. The National Ethics Commission has declared the study “exempt” (the notification letter is attached).

## Funding

 This study was self-funded by the authors and received no external financial support from any funding organization.
